# Decolonising drugs in Asia: the case of cocaine in colonial India

**DOI:** 10.1080/01436597.2017.1357116

**Published:** 2017-08-30

**Authors:** James Mills

**Affiliations:** a Centre for the Social History of Health and Healthcare, University of Strathclyde, Glasgow, UK

**Keywords:** Cocaine, consumption, control, South Asia, colonial, agency

## Abstract

This article examines a drugs trade in Asia that has been largely forgotten by historians and policy-makers, that in cocaine. It will briefly trace some of the contours of this commerce and the efforts to control it. It will also assess how successful these efforts were. The article is designed to contribute fresh perspectives on recent controversies in the historiography of drugs in Asia to argue that the agendas and agency of consumers are central to understanding why markets have formed there for psychoactive substances in the modern period.

## Introduction

After a surveillance operation that had started in January of that year, Bombay Excise Inspector P. J. Hudson decided that he had enough evidence on 7 April 1934 and ordered a raid. The premises in Ghati Gully seemed to be at the centre of an illegal cocaine trade, with the drug stored at one property and sold from another to customers who consumed on the spot after handing over their payment. A posse of officers and constables was dispatched together with an informant and a bogus customer. The plan worked well at first as the ‘customer’ successfully purchased the drug and gave the signal. The posse charged onto the scene and attempted to grab those inside the building only to meet fierce resistance. Gang members responded quickly, and set about the officers with bamboo lathis and broken bottles. The official account in the Bombay Police Gazette noted that ‘the constables, realising the position, undid their belts and started defending themselves. The Inspector and the Sub-Inspector, who were concealed … went to the rescue of the men’. Newspaper accounts were more dramatic, and *The Statesman* reported that ‘overwhelmed by numbers, the small excise party were forced to beat a retreat with four of their men injured’.^1^

This episode points to a drugs trade in south Asia that has been largely forgotten by historians, that in cocaine. The significance of this trade lies in its potential to provide new perspectives on a range of issues in the history of intoxicants, drugs and medicines in Asia. Consumers of intoxicants and narcotics there were certainly the reason for the establishment and early development of today’s international drugs regulatory system, as the 1909 Shanghai Opium Conference lead directly to the establishment of the Opium Advisory Committee and the Permanent Central Opium Board at the League of Nations.^2^ It was Asian consumption of local drugs, opium and to a lesser extent cannabis, which drove these processes and historians have therefore focused on these.^3^ By examining the rapid emergence of a market for cocaine, that most modern of pharmaceutical products in 1900, this article promises to produce a more complex picture of Asia’s drugs consumers. It will challenge accounts written by historians that have assumed that suppliers drove markets, an argument that first emerged in Western imaginations in the nineteenth-century.^4^

To do this, the article therefore also addresses suppliers of cocaine to south Asia in this period. The initial sources of the drug lay outside of Asia, in Holland and Germany, while Japan later established itself as a producer. Answering the question of who transported cocaine into the region will provide important new conclusions about the global circulation of drugs and medicines. In the history of intoxicants in Asia, the focus has been on the supply of opium by Western imperialists to China and East Asia. As recently as 1999, Carl Trocki restated the view that the colonial regime in India, for example, was ‘a global drug cartel which enslaved and destroyed millions and enriched only a few’. Studies by Bello and by Kingsberg have shown that Asian states were equally active, as those in Central Asian kingdoms and in imperial Japan sought to profit from Asian consumers of intoxicants.^5^ The story of cocaine there promises to force a rethink. It is clear that Western colonial governments were quick to prohibit cocaine use in their territories, so the large market in Asia was established despite their policies rather than because of them. The suggestion is that private entrepreneurs, rather than colonial states, became involved in large-scale movements of psycho-active intoxicants and medicines much earlier than the 1940s as previously thought.^6^

A clearer picture of how the market for cocaine was supplied in Asia also promises insights that go beyond the debates about the history of intoxicants there. Studies by Chakrabarti and Attewell of the flow of medicines from Asia have shown that the region had long been the source of medicinal substances for those elsewhere and that exports grew in the nineteenth century.^7^ Other work has suggested that migration from India in this period stimulated the flow of Asian medicines around the world as migrants took their medications with them.^8^ This article provides glimpses of the reverse flow, in order to better understand the arrival of Western medicines in Asia. Much of the work that addresses that process has looked at the impact of state-sponsored colonial medicine in forcing Western ideas and products on local societies as ‘tools of empire’.^9^ By looking at a medicine that arrived in Asia despite the efforts of colonial governments to prevent it, the study promises to reshape ideas so that pharmaceutical companies, medical entrepreneurs and local commercial interests are placed at the heart of accounts of the ways Asia took to Western medicines in the early twentieth-century.

Finally, the story at the start of this article suggests that government struggled to control flows of cocaine. Throughout the nineteenth and early twentieth centuries administrations in Asia lead the way in debating how intoxicant-consuming societies could and should be governed. Historians have argued that their positions were diverse, ranging from efforts to prohibit consumption altogether, to excise regimes that raised revenue from the markets for intoxicants and medicines.^10^ This diversity survived into the twentieth-century and yet by 1910 unanimity had rapidly been established across British territories in Asia that cocaine ought to be prohibited for anything but strictly medical or scientific purposes. Explaining this, and tracing government responses elsewhere in Asia to cocaine in these decades, will provide a clearer and more detailed understanding of a period and a place in which modern systems of governing narcotics markets were established and in which drugs crises were framed for the first time.

## Cocaine consumption in India

In 1902 the *British Medical Journal* printed a report by an Indian doctor, Kailas Chunder Bose, which concluded that:

Besides the use of cocaine hydrochlorate as a therapeutic agent its consumption as a drug for intoxication is so great in Calcutta that unless stringent measures be adopted to control its sale I have reason to fear that its demoralizing effects will soon spread amongst the juvenile members of respectable families and that at no distant date special asylums will be required for the safety and treatment of cocaine inebriates.

He went on to detail 10 cases of cocaine use that he had encountered in the city. The first was L.B.M. ‘a promising boy aged 20, very respectably connected [who] fell into bad company and contracted the habit of taking opium and bhang [cannabis] in their various forms’.^11^ In an effort to give up opium he took to cocaine and before long was taking 30 grains a day (equivalent to about 2 grams). After consuming nothing nutritional but milk he suffered diarrhoea and died. The second case was of a Sanskrit scholar and priest in his forties who was advised by a ‘learned Pundit’ to use cocaine to help him fast. He increased his daily doses and Bose noted that ‘he has given up his priestly duties and mixes freely with low-class people. He lives entirely upon the charity of his neighbours’. Another story merited its own subtitle ‘Cocaine in the Zenana’. Its central figure was ‘a healthy-looking Hindu girl, aged 16 [who] contracted the cocaine habit under peculiar circumstances’:

An elderly woman living in the same house advised her to take cocaine to get rid of dysmenorrhoea which she was subject to. She also cited instances where cocaine proved a sovereign remedy for removing sterility. The foolish girl followed her advice and took cocaine every day clandestinely in 1 gr. doses for six weeks. She then increased the daily dose and one day she took 10 gr [about a half gram]. Half an hour after she had taken the dose she complained of a choking sensation and soon became unconscious. At this stage I was summoned to see her. The patient had all the symptoms of hysteria and I prescribed for her accordingly … at about 10am the following morning marked improvement was noticed in her general condition … at about 1 pm she became very cross and wanted to go to the adjoining room where she had her box containing betel leaves and spices … on opening the folded betel leaf cocaine was discovered and then on being questioned the girl made a clean breast of the whole thing and further said that there were three more girls under the same roof who were taking cocaine in pretty large doses.

The author reported that ‘information has also reached me that women dealing in fancy goods who have access into private houses carry cocaine clandestinely and sell it to girls who take it in small doses with betel leaves’. This was a reference to paan, the popular Indian refreshment in which a number of leaves from the betel creeper are smeared with a variety of ingredients and then formed into triangular shaped parcels. The content of each paan is different according to the preferences of the customer. Common ingredients include areca nut, catechu (from the heart wood of the katha tree) and betel-oil. Once folded into the leaves the parcel is placed in the mouth and chewed. Evidently cocaine had found its way into the list of ingredients.^12^

The *British Medical Journal* was sufficiently intrigued by this that it devoted an editorial piece to the subject. It alleged that ‘the drug is imported in ounce bottles by certain Mohammedan and Marwari dealers. These bottles are sold for 2½ rupees each but the material is more usually put up in small packets costing 1, 2 and 4 pies and retailed by the sellers of betel, the leaf of which is so universally chewed by Indians … unfortunately it appears that some of those addicted to the vice have obtained their supply from dispensaries and even from medical men … the habit is a costly one and this will probably prevent its spread among the lower classes in India’. The editorial concluded that ‘repressive measures must, therefore, be far-reaching as well as drastic if the evil is to be adequately dealt with’.^13^ A half decade later, G. F. Ewens wrote in 1908 that ‘of late years a new habit, that of cocaine taking either by mouth or as snuff or hypodermically has sprung up’. However, he remained convinced that ‘it is an expensive habit and the sufferers are consequently well-to-do and frequently medical men’. As Superintendent of the Lahore Lunatic Asylum his observation was based on patients who had been brought to the hospital.^14^

A report another half decade later painted a different picture of India’s users. Chunilal Bose’s 1913 report to the *British Medical Journal* observed that ‘not many years ago cocaine was a drug hardly known to the people of India outside the medical profession and now, sad to reflect, it has taken a vigorous hold of a certain class of people in this country both in town and village. In Calcutta … there is reason to believe that the cocaine habit has much increased and is rapidly spreading’. Bose had served in the Chemical Examiner’s Department of the Government of Bengal for 27 years, so was well-placed to chart changes in drugs markets as his office was responsible for analysing samples of substances seized by the police and excise officers. His report included details of those though to have died from cocaine consumption thanks to post-mortem reports passed on by a colleague in the police. The first case was B.D., a Hindu male, aged about 23 years, a resident of Calcutta and by occupation a ‘pressman’ [i.e. he worked a press of some sort]. Bose noted that the individual was ‘addicted to alcohol and to cocaine’ before recounting the following story:

On May 28th 1912 he played cards with his friends up to a late hour of the night and distributed pan (betel) with cocaine to his companions taking the largest share himself. He left the place soon after and at 2.30am on May 29th he was found lying unconscious and groaning at a neighbour’s doorway. Medical aid was summoned, the man was removed to hospital, but died on the way there.

Bose noted that the man’s body was poorly nourished and that he detected cocaine in the bowels and urine of the corpse. His second example was of ‘a woman of the town … in the habit of taking cocaine’. Aged about 28 years of age she had left home at 1.30 am and returned five hours later. He reported that ‘she was seen to be staggering while washing her mouth at a hydrant hard by. Very soon afterwards she lay down, became unconscious, and in a few minutes she died’. He noted that there were no signs of violence on her body and that there was a ‘marked quantity’ of cocaine in her viscera.

His final case was of a Hindu female, who lived with her husband in Calcutta, and who was on a visit to her sister-in-law on the day of her death. While there she ‘offered L. some white powder which she believed to be a specific remedy for acidity and indigestion. They each took some of the powder and within half an hour they became ill and then unconscious’. The sister-in-law survived while the young wife died. Bose concluded at the end of these stories that ‘the drug is very easily procurable by the common people [and] the people are getting to be more familiar with its uses’. In little more than a decade after the point that India’s cocaine consumers first came to attention the evidence suggests that drug was being used by both males and females, across the country, and by those of all religions and classes.^15^

The picture remained the same in the 1920s. A. W. Overbeck-Wright was the Superintendent of the mental hospital at Agra. He reported in 1921 that:

The habit is said to have originated in the chawls [working class tenements] of Delhi, being used there first as an application to the glans penis to delay the orgasm and increase the pleasure of the habitués of these places. From this beginning its use has extended till now it is extensively used by debauchees of the worst description.

He included details of two consumers that he had treated in the Agra hospital. The first was a 30-year-old Patwari [keeper of land records] who was ‘wild and excited in appearance and in a continuous state of restless activity’. It seems that he had been admitted by his family and that he had been using cocaine for some time. The Superintendent noted that the man was convinced that ‘his wife and neighbours were in league to poison and electrify him [and] had done him out of his employment’. Overbeck-Wright’s second case was a Muslim syce [groom or stableman] who was aged about 25. The doctor wrote of the patient that ‘he himself had apparently been a vicious debauchee for years, addicted to cocaine and the constant companionship of prostitutes’ and observed that the man was abusive and prone to aural hallucinations. Despite this, however, his case notes suggested that there was some recovery when the patient was denied the drug.^16^

It was suggested that by 1929 ‘somewhere between a quarter and a half a million individuals [were] taking cocaine in India’.^17^ These few anecdotal accounts from the first three decades of the twentieth century do little more than provide glimpses of India’s cocaine consumers, but they do suggest that the market quickly became diverse and complex and remained so. Men and women used the drug, those that did so could be rich or poor, and Muslim and Hindu were numbered among those that sought it. It was consumed for a range of purposes, from the medicinal to the recreational. What is most surprising of all, however, is that this most modern of drugs^18^ was quickly domesticated through that most familiar of modes of consumption in south Asia: paan.

## Control and supply

In 1906 the Council of the Governor General of India met to discuss how controls on cocaine could be imposed across all of the territories under British administration in South Asia. In the notes of the meeting it became clear, however, that efforts to ensure that cocaine could only be used for medical ends dated back to the turn of the century in some parts of India:

Cocaine has been notified as an intoxicating drug within one or other of these Acts since 1900 in Bengal, since 1903 in Bombay and since 1905 in Madras and its sale has been confined in those Provinces to approved chemists, druggists or medical practitioners with the intention that they shall supply it only for medicinal purposes.^19^

The discussion in the council was about extending the policies of these administrations to the whole of the rest of India.^20^ The Excise Act in each of those areas was to be the device used and this meant that cocaine was to be subject to the following restrictions:

No person shall have in his possession any drugs which the Local Government has, by notification … declared to be included in the definition of ‘intoxicating drugs’ except under, and in accordance with the terms of, a general exemption granted by the Local Government.

No person shall have any quantity of any intoxicating drugs … greater than the amount therein specified in respect of such drugs unless he is permitted to collect, cultivate, manufacture or sell the same, or holds a pass therefore from the Collector or some other officer empowered by the Local Government to grant such passes.^21^

Excise Acts were the device by which the colonial government sought to raise revenue by taxing the trade in a variety of products but it was made clear that in the case of cocaine an income was not the intended purpose. The Council of the Governor General of India concluded that in Bombay, Bengal and Madras ‘its sale has been confined … to approved chemists, druggists or medical practitioners with the intention that they shall supply it only for medicinal purposes. It is now most desirable that similar action should be taken … which applies to the rest of India’.^22^ The Bill was approved by the Governor-General on 10th August 1906 and by the India Office back in London on 4 September, so by Autumn 1906 all of the colony was subject to laws that were aimed at suppressing the use of cocaine for anything but medicinal purposes.

It is worth drawing attention to the date, as this action makes the Government of India the first in the world to attempt such a geographically comprehensive ban. In the US individual states imposed restrictions from 1887 onwards^23^ but it was only in 1914 that the Harrison Narcotic Act saw the ‘introduction of national drug prohibition’.^24^ In the UK cocaine possession was first controlled in 1916 through the wartime Defence of the Realm Act 40B but these temporary regulations were placed on a sounder legislative footing only in 1920 with the Dangerous Drugs Act.^25^

When the order from the Council of the Governor-General was presented to the regional governments the message clearly got through. A member of the Legislative Council in the United Provinces, for example, declared that ‘in order to make it clear that in this legislation there is no question of raising revenue I may say that it has been decided that no fees will be taken on licences or passes’.^26^ An Indian member of the same Legislative Council noted that ‘there is no desire on the part of the Government, as I believe is the case, to derive any revenue from the sale of this most injurious drug’.^27^ The President of the Council insisted that ‘the object of this legislation is to put a stop to the cocaine habit … licences would only be given to persons who sell cocaine as a medical drug and the sale of it as an intoxicant would be absolutely prohibited’. Elsewhere he reiterated that ‘the Government proposes to make rules of such stringency as will prevent the sale and possession of cocaine except for bona fide medical purposes’.^28^

While this article started with a story from Bombay in the 1930s that showed that efforts to enforce these rules could be fiercely resisted, it did not take three decades before the authorities realised that their efforts at control were failing. As early as 1907 a letter from the Collector of Customs in Bombay to the Commissioner of Customs, Salt, Opium and A’bkari argued that ‘cocaine is smuggled to a very considerable extent. As the result of searches made at my request by the Postal authorities 44 packages containing cocaine were found in one mail’s delivery at Delhi, and 47 in the registered post between Aden and Bombay … the difficulty of checking its importation is great since it can be conveyed in a very small compass by letter or book-post and a large quantity could be brought as personal baggage by a traveller’.^29^

This letter sparked a nationwide correspondence as the Government of India sought to establish whether this was an isolated case or whether an earlier attempt to prevent cocaine being sent through the post had failed.^30^ The authorities in Burma replied that ‘most of the contraband cocaine which finds its way into Rangoon is brought by the passengers and crews of Chinese steamers plying between Rangoon, Singapore, Hongkong, Swatow, Amoy and other Chinese ports’. Those in Madras reported ‘that large quantities of cocaine pass through the Customs, especially at Calcutta, labelled and invoiced as “powdered Tartaric acid” or mixed with other crystalline substances from which it can be easily separated by solution and obtained pure on recrystallization’ while In Bengal it had been discovered that ‘the seizures made in cases of illicit possession or sale of cocaine, principally in Calcutta, go to show that the bulk of such cocaine is of German manufacture, the principal manufacturer being “Merk”. Preparations also by Messrs. Burgoyne Burbidges and Borrows Welcome [*sic*] of London are also met with from time to time; the proportions of seizures are roughly stated as follows; “Merks” (Germany) 75 percent, Burgoyne’s (London) 15 per cent and Other Makers 10 per cent’.

The Under-Secretary of the Government of the Punjab alleged in 1908 that ‘it would appear that lascars [Indian merchant seamen] are possibly implicated in the contraband importation of cocaine’. Taken together, these letters seem to show that within a couple of years of the nationwide restrictions on cocaine use a number of smuggling routes into India had developed, through the post and the ports, and involving Chinese and Indian sailors.^31^ However, further investigations suggested that it was not just Asian go-betweens who sought to profit from cocaine-smuggling:

Personating a smuggler one of our Excise officers got into dealings with two European members of the crew of a ship recently in Calcutta who appear to be agents of the Glasgow firm in their smuggling trade. One of them instructed the Excise officer how to communicate with the firm and following these instructions the Glasgow firm were asked if they could supply 1000 ozs of cocaine. They have sent a reply offering the cocaine at 25 rupees an ounce … the name of the firm is Messrs Gowan and McLean, St Vincent Street, Glasgow.^32^

Duncan Gowans of the firm was questioned by the police in Scotland and confessed to an elaborate scheme whereby coded messages were sent from Calcutta where he had been supplying Johoor Ali. However, John McGimpsey, the Chief Detective Officer on the case found evidence of a more extensive trade and he concluded that ‘it appears from the correspondence that Mr Wyper of the SS City of Karachi and probably other officials are mixed up with the illegal smuggling of cocaine into India’.^33^

A centralised monitoring system for cocaine was soon established and it began to show how extensive the illicit trade had become. In 1912 in Bombay Presidency over 148 lbs of the drug were seized but in Bengal the figure was 373 lbs. To put this in context legal sales of cocaine in that year amounted to just over 34 lbs in Bombay.^34^ Ten years on the smuggling was still happening. In Madras it was reported that ‘one case of seizure of illicit cocaine occurred during the year. The cocaine was seized on board S.S. Torilla at the Madras Port. The drug was of Japanese manufacture and the quantity amounted to 26418 grains. It was stated that the drug was obtained for sale at Kopio, Japan. The accused in the case consisted of two members of the engine crew, a Chinaman and a Chitagonian’.^35^ In Bombay the authorities seized only 17 lbs while in Bengal they found scarcely more than 1197 ozs. Whether these smaller confiscations point to the success of the Government’s policy or its failure is difficult to say for sure, but it was certainly the case that in some years large seizures indicated a lively traffic in the drug; towards the end of the 1920s Bengal reported that it had confiscated 146 lbs in 1928–1929 compared with 174 lbs in 1927–1928.^36^ Even administrators inland worried about cocaine sales within their territories. In the same year the Government of the United Provinces of Agra and Oudh complained that:

The nefarious traffic in illicit cocaine continued to flourish in spite of the vigilance on the part of the Police and Excise staffs and the heavy sentences which were generally passed in the cases which reached the courts. Most of the bigger cities were affected by the evil and the cocaine habit was undoubtedly wide-spread among the urban population. Very few cases were reported from rural tracts. Illicit cocaine came into the country from Germany, Japan and Holland; and smugglers in these provinces got their supplies from Calcutta, Bombay, Delhi and Ajmere. Detection was not an easy matter. The drug being odourless and small in bulk was therefore easily smuggled. The principals engaged in the business seldom handled the drug which was hawked by hirelings for sale to the public. It was seldom therefore that organisers were caught and as the profits were large smugglers spent money readily and experienced no difficulty in securing the services of undesirable persons as carriers and agents.^37^

Clearly then, smuggling of cocaine to India continued during and after the First World War and in spite of the international control system set up in 1919.^38^ However, after the First World War more cocaine seemed to be coming from Japan. This was demonstrated in a report prepared for the Government of India in 1931 by J. Slattery of the Central Board of Revenue. He noted that ‘in 1929 particular attention was drawn to it [cocaine] by reason of several large seizures each of more than a thousand ounces, made at Calcutta. The total quantity seized in that year in Calcutta and Rangoon came to some 9000 oz and if that figure represented five per cent of the whole the total illicit import reached the alarming figure of 180000 oz or 11250 lbs’.^39^ The cocaine seized carried the labels of the Fujitsuru, Buddha and Elephant brands but it was discovered that no legitimate pharmaceutical company produced these. The seizures were made on board ships of the British India Far East Company and the Indo-China Line of the Jardine-Matheson group. Given this information, Slattery headed for Japan and China via Singapore and Hong Kong. Conducted between January 1930 and July 1931, his survey reported corrupt officials, secretive police officers and shadowy organisations. He concluded that:

The Chinese and Indians on the British India steamers received their supplies from different sources, the Chinese from Kwong Yee Sang etc. [at Kobe] and the Indians from Japanese traffickers, the Chinese obtaining the Fujitsuru brand and the Indians the various Japanese brands. Also seeing that the Fujitsuru brand was represented in India as being of German manufacture and so commanded a better market than the Japanese article the sources supplying the Fujitsuru variety were not open to the Indians.^40^

Slattery, however, was not telling the full story here as he had not just come across Chinese and Indian smugglers. A couple of years earlier he interviewed a British sailor named Gibson who had been arrested in Rangoon. Gibson openly admitted that ‘I traded on the fact that as a British ship’s officer I was above suspicion’^41^ and he exploited this by purchasing cocaine in Japan and selling it in India.^42^ Nevertheless, Slattery’s investigation clearly shows that three decades of colonial regulations designed to ‘put a stop to the cocaine habit’ had succeeded only in creating complex networks that provided an illicit supply of the drug to south Asian consumers.

## Conclusion

Why is the above story important for those considering the history of intoxicants, drugs and medicines in Asia? The answers lie in the challenge it presents to accounts of consumers of such substances there that have lingered ever since the nineteenth-century. As debates raged in Britain about the First Opium War, a trope emerged in which critics of the country’s actions in China conjured up images of Asian opium consumers as hapless dupes of wicked British opium suppliers. The Reverend Algernon Thelwall, for example, asked indignantly in 1839 how the British government could ‘stand by unconcerned, and countenance, in its enlightened and professedly Christian subjects, that system of smuggling, by which a poisonous drug is introduced into China to the ruin and destruction, moral and physical, of thousands and tens of thousands of its inhabitants’.^43^ Jump forward to more recent times and Carl Trocki remains convinced that the British who traded opium between their possessions in India and the Chinese empire were ‘a global drug cartel which enslaved and destroyed millions and enriched only a few’^44^ while Martin Booth states simply that ‘inevitably, as the availability of opium rose so did the demand for it’.^45^ In between times the whole international drugs regulatory system was established in order to deal with the ‘problem’ of Asian consumption of opium and opiates. Supply drove demand was the underlying logic; as wily Westerners supplied, helpless Asians consumed.

Post-colonial studies provided the tools to challenge these assessments. Following Edward Said’s work, scholars were more alert to the idea that easy generalisations in which Westerners were dominant and determined and Others simple and weak had their origins in the myth-making projects of colonial-era Orientalism.^46^ Those influenced by the Subaltern Studies project began to study more closely the agendas and agency of groups marginalised by the colonial state and local elites and found an ‘autonomous domain’ in which ideas and actions often drew on neither.^47^ Work on commoditization and its cultural history was also important as in drawing attention to ‘the social life of things’^48^ historians and anthropologists argued that individuals ‘with their drive to discriminate, classify, compare and sacralize’ could be active agents in attaching values to commodities.^49^

Among the first historians to provide revisionist accounts were Frank Dikötter, Lars Laamann and Zhou Xun, who challenged the idea of a nineteenth-century ‘opium plague’ in China as the product of the colonial era. They argued that it was the invention of Christian missionary’s keen to conjure up a moral crusade to energise support for their efforts among the Chinese. It has proven remarkably durable as the Communist Party (CCP) seized on the idea that Western countries had poisoned their way to power in China as a powerful ideological image. It also featured heavily in the early stages of the establishment of the international drugs regulatory framework where US interests deployed it. In researching recently opened Chinese archives, Dikotter, Laaman and Zhou instead found sophisticated consumer cultures around opium. Different grades and a range of preparations of opium were on offer, and imported versions from India or Central Asia vied for customers with the domestic product produced across China. The elites could afford to sample whatever was on offer and incorporated their favourite types into both social events and private moments of relaxation. Others tailored their tastes to their incomes, and consumers varied from those that took opium on a daily basis to others who would try a little only at gatherings or when in company. For much of the nineteenth-century and into the twentieth, taking preparations of opium was generally considered socially acceptable in China.^50^ Yangwen Zheng found similar evidence and concluded that ‘opium smoking could not have come to a better place at a more opportune moment; it was a welcome addition to the Ming–Qing–Republican economy, culture and society’.^51^ Such accounts ‘decolonised’ drugs histories in Asia by seeking to move beyond colonial-era images and constructs.

The story about cocaine in South Asia similarly challenges these colonial-era ideas. There is no well-organised supplier of the drug that might be accused of acting as the East India Company may have done with opium in China. Instead, what emerges from the records is a diverse and disparate range of opportunists who sourced batches of the drug to smuggle into India to take advantage of the prices inflated by British controls. Cocaine was produced by only a limited number of companies at this time, so the labels of such corporations as Merck and Wellcome Burroughs were often found on seized consignments. However, this was not because those corporations were themselves organising a market for their products in India, but rather because smugglers were simply buying up legal supplies in Europe (where cocaine was largely unregulated until 1917) for illegal profits in British territories in south Asia. The story of the Glasgow chemist and his Indian partners is the clearest demonstration of this tangle of relationships, as the source of the drug was a local shop in Scotland which had been approached by imperial sailors at the bidding of Calcuttans eager to exploit the market in Bengal. It is true that after the First World War Japan and its colonies seem to have become a well-organised and state directed source of cocaine.^52^ By then the supplier was simply filling a gap in the Indian market left by the disruption and decline of European supplies due to the hostilities of the period. It was acting as a supplier to an existing market rather than as the force behind a new one.

If the story fails to provide any evidence of a core supplier acting consciously to create a new market it certainly does offer glimpses of a rapidly growing and increasingly powerful market. It is the complexity of the latter that is most striking and which resonates so strongly with other examples where a drug has suddenly infiltrated a society. The domestication of cocaine through its incorporation into the south Asian rituals of taking paan is significant and striking. It seems that Indians may have developed an appreciation of that most modern of drugs, but that it did so only through a traditional mode of consumption. The sources show that cocaine may well have been recommended in the first place as a medicine, and that it retained a reputation as a therapeutic thereafter. It was also purchased as an aid to recreation and relaxation and as a tonic, a pick-me-up that falls somewhere between medication and intoxication. It was consumed across India’s various communities and throughout the country, in a range of social contexts, and by both genders. It sometimes appears as a solitary pursuit but often was consumed in company. Crucially this market developed despite the efforts of colonial governments to prohibit it. In India, regulation had been in place since 1900 and yet the story that opened this article shows that over three decades later both controls and enforcement mechanisms were resisted, sometimes violently.

In all of this the cocaine market in south Asia resembles opium in nineteenth century China, where the substance ‘went from medicine to mass drug food [and] patterns of consumption altered, demand increased and the understanding of opium use changed’.^53^ It also resembles *yaa*-*baa* in contemporary Thailand. There the drug was originally used as an aid to productivity by workers, but now ‘consumption is indeed extremely diverse and wide-ranging, and the drug in turn proves able to satisfy the expectations of many different types of users’.^54^ Among the latter can still be found labourers and office-staff, but these have been joined by groups drawn from throughout society including elite school and university students, street-children and prostitutes.

It is the Asian consumer that seems to be driving the market in all of these instances. Experimentation and innovation ensure that substances with initially limited functions, but with enticing properties, are quickly adopted for a wider set of purposes. In the examples from China and Thailand, this experimentation and curiosity on the part of individual consumers seems to be linked to social, cultural and economic change, and it was certainly the case that the decades considered here in which cocaine spread south Asia India were marked by ‘severe dislocations’.^55^ None of this is to deny that suppliers have a role in the development and expansion of markets once they exist, and historians are right to insist that ‘one has to provide a clearer picture of the push and pull factors that are so characteristic of an illegal economy’^56^ when considering the consumption of illicit drugs. It is, however, to challenge the assumptions and assertions of those who look chiefly to Western suppliers in explaining the origins of markets for intoxicants in Asia in the modern period. Cocaine may have been a thoroughly modern Western drug, the product of late nineteenth century laboratory science and of the technological processes of the maturing pharmaceutical industry as it entered the 1900s. But it was Indians who incorporated it into their rituals of consumption, their medical practices and their tastes for intoxicants, and who thereby turned south Asia into one of the largest markets for cocaine of the twentieth century. It is this conclusion that adds to the recent historiographical push to decolonise drugs and drugs consumers in Asia and to recover the agendas and agency of those using psychoactive substances there in order to provide a clearer view of the forces driving markets for them.

## Disclosure statement

No potential conflict of interest was reported by the author.

## Funding

This work was supported by the Wellcome Trust [grant number WT200394/Z/15/Z].

## Note on Contributor

***James Mills*** is Professor of Modern History at the University of Strathclyde and was founding Director of the Centre for the Social History of Health and Healthcare (CSHHH) Glasgow. His publications include *Cannabis Nation: Control and Consumption in Britain, c.1928–2008* (Oxford University Press, 2012), *Cannabis Britannica: Empire, Trade and Prohibition, 1800–1928* (Oxford University Press, 2003) and the edited volume (with Patricia Barton) *Drugs and Empires: Essays in Modern Imperialism and Intoxication* (Palgrave Macmillan, 2007). His is currently PI on the Wellcome Trust funded project ‘The Asian Cocaine Crisis: Pharmaceuticals, consumers and control in South and East Asia, c. 1900–1945’ (see www.strath.ac.uk/cshhh).

## Note on Sources

IOL refers to files from the India Office Library at the British Library in London; NA signifies files from the UK’s National Archives in Kew; Bombay General Files can be found in the Maharashtra State Archives in Mumbai.

## Notes

1. India Office Library (British Library) IOL File L/I/1/203, ‘Excise Supplement to the Bombay Police Gazette 28 February 1935’.

2. Bruun et al., *The Gentlemen’s Club*; McAllister, *Drug Diplomacy*.

3. Trocki, *Opium, Empire and Political Economy*; Mills, *Cannabis Britannica*.

4. Booth, *Opium*; see also Travis Hanes and Sanello, *The Opium Wars.*

5. Bello, *Opium and the Limits of Empire*; Kingsberg, *Moral Nation.*

6. Chouvy and Meissonnier, *Yaa Baa.*

7. Chakrabarti, *Materials and Medicine*; Attewell, “Interweaving Substance Trajectories.”

8. Mills, “Globalising Ganja.”

9. Headrick, *Tools of Empire.* The exception is Nandini Bhattacharya’s recent article which explores the multiple routes by which Western medicines found their ways to Indian markets; Bhattacharya, “Between the Bazaar and the Bench.”

10. For a sense of the diversity of these efforts see the essays in Mills and Barton, Drugs and Empires.

11. Opium and cannabis (‘bhang’) had been popular sources of medication and intoxication for centuries in south Asia. For details see Richards, “Opium and the British Indian Empire”; Mills, *Cannabis Britannica*.

12. Extracts taken from Chunder Bose, “Cocaine Intoxication and its Demoralizing Effects.”

13. ”Cocainism in Calcutta,” 1041–1042.

14. Ewens, *Insanity in India*, 116–118.

15. Bose, “Cocaine Poisoning,” 16–17.

16. Overbeck-Wright, *Lunacy in India,* 271–273.

17. Chopra and Chopra, *Cocaine Habit in India*, 1022.

18. The alkaloid ‘cocaine’ was only isolated in 1860 by the German Albert Niemann and its chemical formula described in 1862; Berridge, *Opium and the People*, 217. For an overview of its manufacture see Courtwright, *Forces of Habit*, 50.

19. IOL L-PJ-6–776, File 2878, note 1.

20. The areas named are the United Provinces of Agra and Oudh, the Punjab, the North-West Frontier Province, the Central Provinces, Burma, Coorg and Ajmer.

21. IOL L-PJ-6–776, File 2878, note 2.

22. IOL L-PJ-6–776 File 2878, note 6.

23. Streatfeild, *Cocaine*, 136.

24. Spillane, *Cocaine*, 1.

25. Berridge, *Opium and the People*, 251–264.

26. IOL L/PJ/6/747 File 428, note 3.

27. IOL L/PJ/6/747 File 428, note 4.

28. IOL L/PJ/6/747 File 428, note 5.

29. IOL R-20-A-1437, File 54/1, note 12.

30. IOL R-20-A-1437, File 54/1, note 13.

31. IOL R-20-A-1437, File 54/1, note 14.

32. UK National Archives (NA) HO 45/10601/189271, note 19.

33. NA HO 45/10601/189271, note 20.

34. IOL V-25–323-13, note 15. Figures for legal sales in Bengal are not available for 1912/13.

35. IOL V-25–323-13, note 16.

36. IOL V-25–323-13, note 17.

37. Ibid.

38. McAllister, *Drug Diplomacy*, 37.

39. Slattery, *Investigation of the Problem of Smuggling*, 1.

40. Ibid., 155–156.

41. NA FO 371/14768/248.

42. Meyer and Parssinen, *Webs of Smoke*, 121–124.

43. Thelwall, The iniquities of the opium trade with China, 72.

44. Trocki, *Opium, Empire and Political Economy.*

45. Booth, *Opium.*

46. Said, *Orientalism*.

47. Guha, *Subaltern Studies,* 4.

48. Appadurai, *Social Life of Things*.

49. Kopytoff, “Cultural Biography of Things,” 87.

50. Dikötter et al., *Narcotic Culture.*

51. Yangwen, *The Social Life of Opium in China, 1483–1999*, 9.

52. Karch, “Japan and the Cocaine Industry,” 154–158.

53. Brook and Tadashi Wakabayashi, *Opium Regimes*, 7.

54. Chouvy and Meissonnier, *Yaa*-*Baa*, xx.

55. Bose and Jalal, *Modern South Asia*, 126.

56. Chouvy and Meissonnier, *Yaa*-*Baa*, xix.
